# Pretherapeutic ^18^F-PSMA PET/CT Reveals Incidental Tracheal Epithelial–Myoepithelial Carcinoma

**DOI:** 10.3390/diagnostics16060883

**Published:** 2026-03-16

**Authors:** Farid Gossili, Nelson Fuentes-Martinez, Christian Høyer

**Affiliations:** 1 Department of Clinical Physiology, Viborg Regional Hospital, 8800 Viborg, Denmark; 2 Department of Clinical Medicine, Aalborg University, 9220 Aalborg, Denmark; 3 Department of Pathology, Aarhus University Hospital, 8200 Arhus, Denmark; nelmar@rm.dk

**Keywords:** ^18^F-PSMA PET/CT, atypical intense PSMA uptake, tracheal tumor, epithelial–myoepithelial carcinoma, non-prostatic neoplasms

## Abstract

A 75-year-old man with newly diagnosed high-risk prostate cancer (cT3bN0M0) underwent ^18^F-PSMA PET/CT, which demonstrated intense tracer uptake in a left tracheal mass causing near-complete luminal obstruction, raising suspicion of a primary lung malignancy or metastatic disease. Endoscopic debulking was performed due to progressive respiratory symptoms with dyspnea. Histopathology and immunohistochemistry (p63, SMA, CK5/6 positive; PSA, NKX3.1, and AR negative, with downregulated PSMA-expression) established the diagnosis of low-grade epithelial–myoepithelial carcinoma of the trachea. Following debulking, the patient’s symptoms resolved, and a watchful-waiting strategy was adopted for the tracheal tumor, while curative-intent therapy for prostate cancer continued. This case highlights that ^18^F-PSMA PET/CT may reveal rare, intensely PSMA-avid non-prostatic neoplasms and underscores the importance of recognizing atypical uptake patterns to avoid misinterpretation during prostate cancer staging.

**Figure 1:**
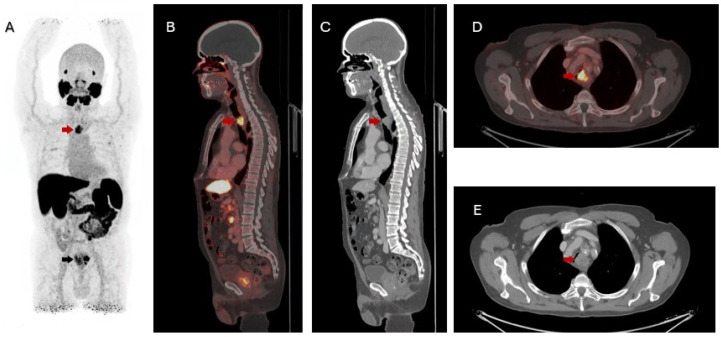
Pretherapeutic ^18^F-PSMA PET/CT of a 75-year-old man with high-risk prostate cancer (biopsy confirmed typical adenocarcinoma, Gleason 9 [4+5], cT3bN0M0 and PSA 26.1 ng/mL) in maximum intensity projection (**A**) shows intense uptake in the prostate (SUVmax 29, black arrow) and a focal tracheal lesion (SUVmax 9.1, red arrow), corresponding to a miPSMA score of 2 based on PROMISE criteria. Clinically, the patient had progressive respiratory symptoms, including dyspnea and reduced exercise tolerance. Sagittal fused PET/CT (**B**) and contrast-enhanced CT (**C**) demonstrate a 2.9 × 2.2 cm left lateral tracheal mass at Th2–3. Axial fused PET/CT (**D**) and CT (**E**) reveal near-complete luminal obstruction and possible mediastinal and esophageal involvement. The findings raised suspicion for a primary lung malignancy versus metastasis; due to the lack of nodal or osseous metastases, a non-prostatic pathology was suspected, and a biopsy was recommended.

**Figure 2:**
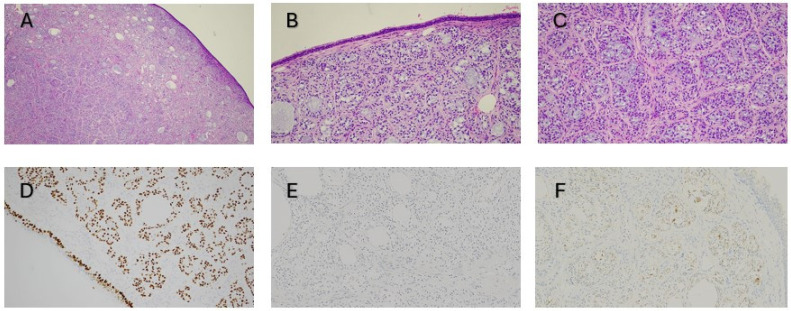
Histopathology of the tracheal mass after endoscopic debulking utilizing hematoxylin and eosin (H&E) staining shows a well-circumscribed tumor respecting the normal respiratory epithelium, with cystic dilatations and islands of vacuolated cells (×4: (**A**); ×10: (**B**) and (**C**)). Immunohistochemistry revealed a biphasic tumor with myoepithelial predominance. P63 (×10: (**D**)), S100, SMA, and CK5/6 were positive in the myoepithelial component, while CD117 and CK7 were positive in the epithelial component. PSA (×10: (**E**)), NKX3.1, AR, and TTF-1 were negative. PSMA staining (×10; (**F**)) showed weak, granular cytoplasmic positivity in the tumor cells, consistent with downregulation of PSMA expression. The Ki-67 index was ~4%, with no necrosis or mitoses. Next-generation sequencing showed no pathogenic variants or gene fusions, confirming a low-grade epithelial–myoepithelial carcinoma. Post-debulking, symptoms resolved; surveillance was chosen for the tracheal tumor while curative-intent radiotherapy was chosen for the prostate cancer. ^18^F-PSMA PET/CT may reveal incidental, intensely PSMA-avid non-prostatic neoplasms due to PSMA expression in the tumor neovasculature [1]. Furthermore, PSMA expression has been demonstrated in human salivary gland tissue by cytoplasmic immunohistochemical staining of the epithelium of acinar glandular cells [2], which explains the physiological uptake seen on PSMA PET scans. Therefore, PSMA uptake can also be expected in tumors originating from epithelial cells, as previously described in adenoid cystic carcinoma in case reports [3,4] and preliminary analyses exploring the use of PSMA PET in the management of these tumors [5]. However, other very rare epithelial-derived salivary gland tumors, such as epithelial–myoepithelial carcinoma, have not yet been described to the best of our knowledge. Because PSMA-expression may be either down- or upregulated in different tumor types [5], this report may be relevant for future consideration of potential PSMA PET applications in such tumors. Our case demonstrated pathological downregulation of PSMA-expression in tumor cells compared with normal epithelial cells, yet it showed intense uptake in PSMA PET. In addition, awareness of atypical PSMA uptake patterns is crucial to prevent misinterpretation, especially during primary staging of prostate cancer. This case further highlights a rare tracheal epithelial–myoepithelial carcinoma mimicking metastatic prostate cancer in PSMA imaging, emphasizing the potential role of PSMA PET in detecting epithelial cell–derived tumors.

## Data Availability

The original contributions presented in this study are included in the article. Further inquiries can be directed to the corresponding author.
